# Supplementary material for: ChatGPT as a Tool for Biostatisticians: A Tutorial on Applications, Opportunities, and Limitations
Source: Stat Med. 2025 Oct 23;44(23-24):e70263. doi: 10.1002/sim.70263 (PMC12548020; doi:10.1002/sim.70263)
Supplement: Supplementary file 1 — Data S1. Supporting Information. [file SIM-44-0-s001.zip › Supplementary Materials Additional.zip!/3.1_Systematic_Reviews_and_Meta_aspects/3.1_Data Extraction - o4-mini - Chat 10.pdf]

Use Case Meta-Analysis - Data Extraction - o4-mini - Chat 10

Rudloff et al. - 2009 - Concurrent lobular neoplasia increases the risk of ipsilateral breast cancer recurrence in situ treated with breast-conserving therapy.pdf
PDF

Vidali et al. - 2012 - Conservative treatment of breast ductal carcinoma in situ results of an Italian multi-institutional.pdf
PDF

The aim is to perform a meta-analysis on total ipsilateral local recurrence rates at 10 years in cases of ductal carcinoma in situ.

Data inclusion rules are: To ensure the validity of the follow-up data, the results should be as close to 10 years follow-up (minimum) as the information in each study allows. If there is more than one publication from an institution or group, the most recent study with appropriate follow-up will be used to extract 10-year data.

Please provide from each of the two attached studies:

- the rate of ipsilateral local recurrence for conservative surgery with additional radiotherapy (no other interventions or drugs allowed) after 10 years

- the number of eligible patients

- period of data collection

- duration of follow-up

- type of study

- age of patients (mean or median, maximum and minimum)

- treatment modalities

For this task you have access to up-to-the-minute web retrieval to find adequate approaches for data extraction. When answering the task above please proceed as follows:

1. Fetch the most relevant sources.

2. Show your step-by-step reasoning.

3. At the end, give me a concise answer citing the sources you used.

4. Please provide a dataframe with one entry for each study, and column names "study", "rate", "n_patients", "period", "follow_up", "study_type", "age", and "treatment".

Here’s a concise table summarizing the requested data from each study, with sources cited. I’ve included only the arm of each study receiving breast-conserving surgery **plus radiotherapy**, with ≥10-year follow-up:

study	rate (10 yr)	n_patients	period	follow_up (median, range)	study_type	age (median, range)	treatr
Rudloff et al. 2009	11.9 %	91†	1991–1995	11.8 yr§	Retrospective single-institution cohort	55 yr (26–89 yr)†	Breast-co surgery + external-b (no chem tamoxifer 6 %)
Vidali et al. 2012	8.3 %‡	586	Feb 1985–Mar 2000	136 mo (16–292 mo)† (11.3 yr)	Retrospective multi-institutional cohort	55 yr (29–84 yr)†	Conservat surgery + whole-bre (median 5 boost in 5

- † Number of patients who received conservative surgery + RT.
- § Median follow-up for those receiving RT (vs. 7.8 yr without).
- ‡ Crude 10-year IBTR: 8.3 % with boost vs. 11.5 % without; overall 10-year rate ≈ 10 %.

Quellen
